# Clinical and Nutritional Management of Gallbladder Mucocele in Dogs: A Four-Case Series

**DOI:** 10.3390/ani16172771

**Published:** 2026-09-03

**Authors:** Natália M. C. Oliveira, Pedro H. Marchi, Leonardo A. Príncipe, Andressa R. Amaral, Gabriela P. T. Moreno, Laís O. C. Lima, Natacha Teixeira, Maria C. F. Pappalardo, Nicolle P. N. Gonçalves, Júlio C. C. Balieiro, Carla G. Vecchiato, Thiago H. A. Vendramini

**Affiliations:** 1 Veterinary Nutrology Service, Veterinary Teaching Hospital, School of Veterinary Medicine and Animal Science, University of Sao Paulo, Sao Paulo 05508-270, Brazil; nataliamanuela@usp.br (N.M.C.O.); andressa.rodrigues.amaral@alumni.usp.br (A.R.A.); gabrielapinheirotm@gmail.com (G.P.T.M.); laisocl@usp.br (L.O.C.L.); natachateixeira@alumni.usp.br (N.T.); carolpappalardo@gastropet.com.br (M.C.F.P.); nicollegoncalves@usp.br (N.P.N.G.); 2 Pet Nutrology Research Center, Department of Animal Nutrition and Production, School of Veterinary Medicine and Animal Science, University of Sao Paulo, Pirassununga 13635-900, Brazil; pedro.henrique.marchi@usp.br (P.H.M.); leoprincipe@usp.br (L.A.P.); balieiro@usp.br (J.C.C.B.); 3 Department of Veterinary Medical Sciences, University of Bologna, via Tolara di Sopra 50, 40064 Ozzano Emilia, Italy; carla.vecchiato2@unibo.it

**Keywords:** biliary disease, dietary management, hyperlipidemia, nutrition

## Abstract

Gallbladder mucocele is a common extrahepatic biliary condition in dogs, traditionally managed with cholecystectomy, while conservative approaches combining clinical and nutritional strategies remain underexplored. This case series reports four dogs diagnosed with gallbladder mucocele, all presenting with hyperlipidemia, three with overweight or obesity, and one with hypothyroidism. The dogs were treated using a multimodal protocol combining pharmacological therapy with nutritional strategies. The pharmacological protocol included ursodeoxycholic acid, silymarin, S-adenosyl-L-methionine, bezafibrate, and omega-3 fatty acids, with doses adjusted according to body weight. Nutritional interventions were individualized and included weight-loss diets and a low-fat gastrointestinal diet. Clinical and ultrasonographic outcomes varied among cases, with partial or complete resolution observed over different follow-up periods. These findings suggest that clinical management combined with individualized nutritional protocols may be a potential approach for asymptomatic dogs with gallbladder mucocele, particularly when surgery is not feasible, and could inform future investigations.

## Simple Summary

Gallbladder mucocele is a common disease affecting the gallbladder in dogs and is often treated with surgery. However, non-surgical management options are still not well understood. This study describes four dogs with this disease that were treated with a combination of medications and individualized dietary interventions. Different feeding strategies were used, including weight-control and low-fat diets, depending on each dog’s needs. All dogs showed improvement over time, with partial or complete resolution of the ultrasonographic features of GBM. These findings suggest that combining medical treatment with appropriate nutrition may be a useful alternative for managing this condition, especially in early or asymptomatic cases or when surgery is not possible. Overall, this study raises hypotheses regarding the importance of personalized dietary strategies in the management of dogs with gallbladder disease.

## 1. Introduction

Gallbladder mucocele (GBM) is a disorder characterized by the abnormal accumulation of bile and mucus within the gallbladder due to increased mucin secretion, leading to luminal distension with immobile, adherent content [1,2,3]. This condition is currently recognized as one of the main extrahepatic biliary diseases in dogs, with an increasing number of diagnoses reported in clinical veterinary practice [1]. The clinical presentation is often nonspecific, and the condition may be incidentally diagnosed in asymptomatic patients [4]. However, as the disease progresses, more evident clinical signs may develop, particularly in cases complicated by gallbladder infarction, biliary obstruction, or rupture. In these cases, common manifestations include vomiting, abdominal pain, lethargy, jaundice, and fever [5].

Given the potential for disease progression, early diagnosis is essential for appropriate clinical decision-making. Abdominal ultrasonography remains the primary diagnostic tool, with characteristic findings such as stellate or finely striated bile patterns and reduced gallbladder motility supporting the diagnosis of GBM [6,7]. In addition to imaging findings, clinicopathological parameters contribute to the assessment of disease severity and associated metabolic alterations. Hyperbilirubinemia is more frequently observed in advanced cases, particularly in the presence of biliary obstruction or rupture [8], while increases in alkaline phosphatase (ALP) and alanine aminotransferase (ALT) reflect hepatobiliary involvement [3]. Furthermore, hypercholesterolemia and hypertriglyceridemia are commonly reported and may be associated with the metabolic disturbances underlying GBM development.

Cholecystectomy is currently considered the treatment of choice, particularly in symptomatic or advanced cases. However, surgical intervention may not be feasible in all patients because of clinical instability, comorbidities, or owner-related factors. Given that dietary factors can influence bile acid composition and gallbladder motility, and that high-fat intake has been associated with impaired motility and alterations in bile composition, conservative management, particularly when combined with nutritional strategies, may represent a viable option in asymptomatic patients or poor surgical candidates [8,9,10]. However, although partial or complete resolution of GBM has been reported with conservative management [11,12], this approach remains controversial due to the limited number of documented cases and the variability among treatment protocols.

Retrospective studies suggest higher survival rates in surgically treated dogs compared to those managed medically [13]. However, these findings are often limited by the lack of standardized clinical and nutritional approaches. Dietary interventions are frequently described in a nonspecific manner, typically restricted to general “low-fat” recommendations, without consideration of the metabolic context in which the disease develops. As a result, the potential role of individualized pharmacological and nutritional strategies in conservative management remains poorly characterized and warrants further investigation. This case series describes four dogs with GBM managed through a combined pharmacological and nutritional approach, emphasizing clinical and ultrasonographic outcomes and illustrating practical considerations for non-surgical management.

## 2. Case Report Series

Four dogs diagnosed with GBM based on characteristic ultrasonographic findings (immobile, non-gravity-dependent gallbladder contents with a striated or stellate pattern, which also aided in differentiating GBM from biliary sludge) [1,6,7] were managed using a combined pharmacological and nutritional approach. The pharmacological treatment was standardized across cases to maintain consistency in the clinical management of the case series, whereas nutritional strategies were individualized according to each patient’s clinical and metabolic needs. The cases included patients with different clinical presentations and metabolic profiles, including obesity, hyperlipidemia, and other endocrine disorders, commonly reported in association with GBM.

Before the diagnosis of GBM, the dogs were fed different commercial diets according to their previous clinical conditions. The nutritional composition and main ingredients of these pre-diagnosis diets are presented in Table 1. However, as the patients had not yet undergone nutritional assessment or received individualized recommendations, daily nutrient and energy intakes before diagnosis could not be determined.

Following the diagnosis of GBM, dietary interventions consisted of a homemade weight-loss diet (Case 1), a commercial weight-loss diet (Cases 2 and 3), and a commercial low-fat gastrointestinal diet (Case 4). The dietary compositions are presented in Table 2, and the daily nutrient and energy intakes are presented in Table 3.

### 2.1. Case 1

The first case, an eight-year-old female Pug, was referred for specialized endocrine evaluation due to suspected hypothyroidism. The patient had a chronic history of recurrent *Malassezia* otitis, generalized lethargy, and reduced activity levels, partially attributed to concomitant orthopedic conditions, specifically metacarpal and spinal joint pain, which were being monitored. Upon physical examination, the patient weighed 8.3 kg and was classified as overweight, with a body condition score (BCS) of 7/9 [16]. Notably, despite the elevated adiposity, mild muscle wasting was observed, with a muscle mass score (MMS) of 2/3 [17].

**Figure 1:**
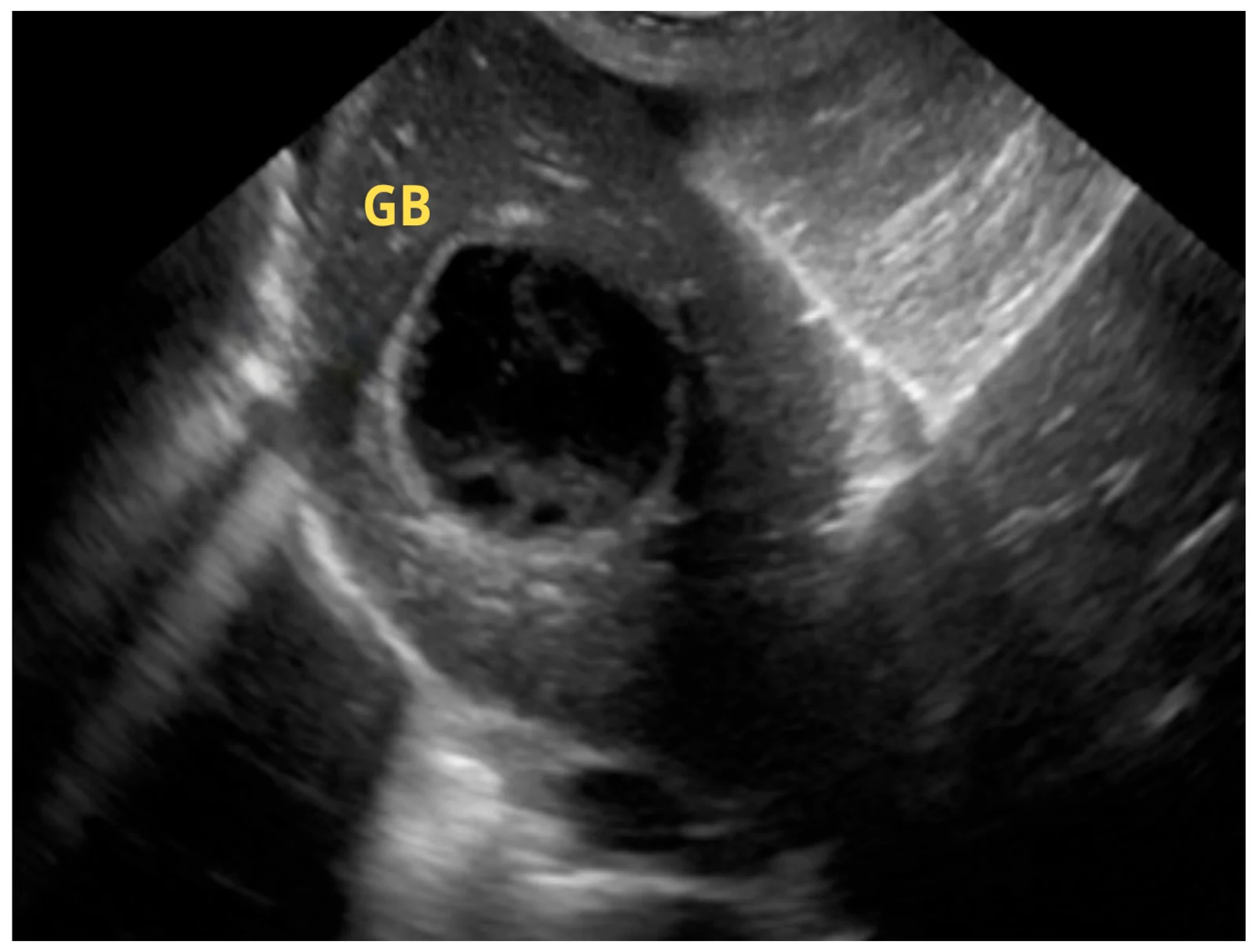
Abdominal ultrasonography of the gallbladder (GB) in an 8-year-old female Pug (Case 1). The gallbladder is distended and contains immobile, hyperechoic intraluminal material forming a stellate (“starry”) pattern, consistent with gallbladder mucocele (GBM).

To investigate the suspected hormonal disorder, thyroid function tests were performed, including measurement of thyroid-stimulating hormone (TSH) and free thyroxine (T4) by dialysis. Initial laboratory screening revealed significant metabolic alterations, including a marked elevation in ALP activity at 468 U/L (23–212 U/L) and severe hypertriglyceridemia at 324 mg/dL (50–100 mg/dL). Other hematological and biochemical parameters were within normal limits and are presented in Supplementary Table S1. Follow-up abdominal ultrasonography revealed a distended gallbladder containing immobile, echogenic, stellate-patterned intraluminal content, consistent with GBM (Figure 1).

Following the ultrasonographic diagnosis of GBM and given the absence of clinical signs or laboratory evidence of disease progression, combined with the owner’s preference to avoid surgical intervention, a conservative clinical management protocol was initiated. The pharmacological strategy was designed to provide choleretic, hepatoprotective, and hypolipidemic effects, and consisted of ursodeoxycholic acid (UDCA) (15 mg/kg), silymarin (20 mg/kg), S-adenosyl-L-methionine (SAMe) (20 mg/kg), bezafibrate (5 mg/kg), and omega-3 fatty acids (120 mg of EPA and DHA/kg BW^0.75^), all administered orally once daily.

Concurrently, a customized homemade weight-loss diet was formulated. The daily ERWL was calculated based on the patient’s target body weight (TW), defined as 15% below the initial body weight (7.05 kg), following the protocol described by Brooks et al. (2014) [14]. The metabolic energy was determined using the following equation: ERWL = 70 × (ideal body weight)^0.75^. The diet was specifically designed to address both obesity and biliary stasis. Clinical and ultrasonographic reassessment was scheduled 30 days after the initiation of the combined pharmacological and nutritional protocol.

**Figure 2:**
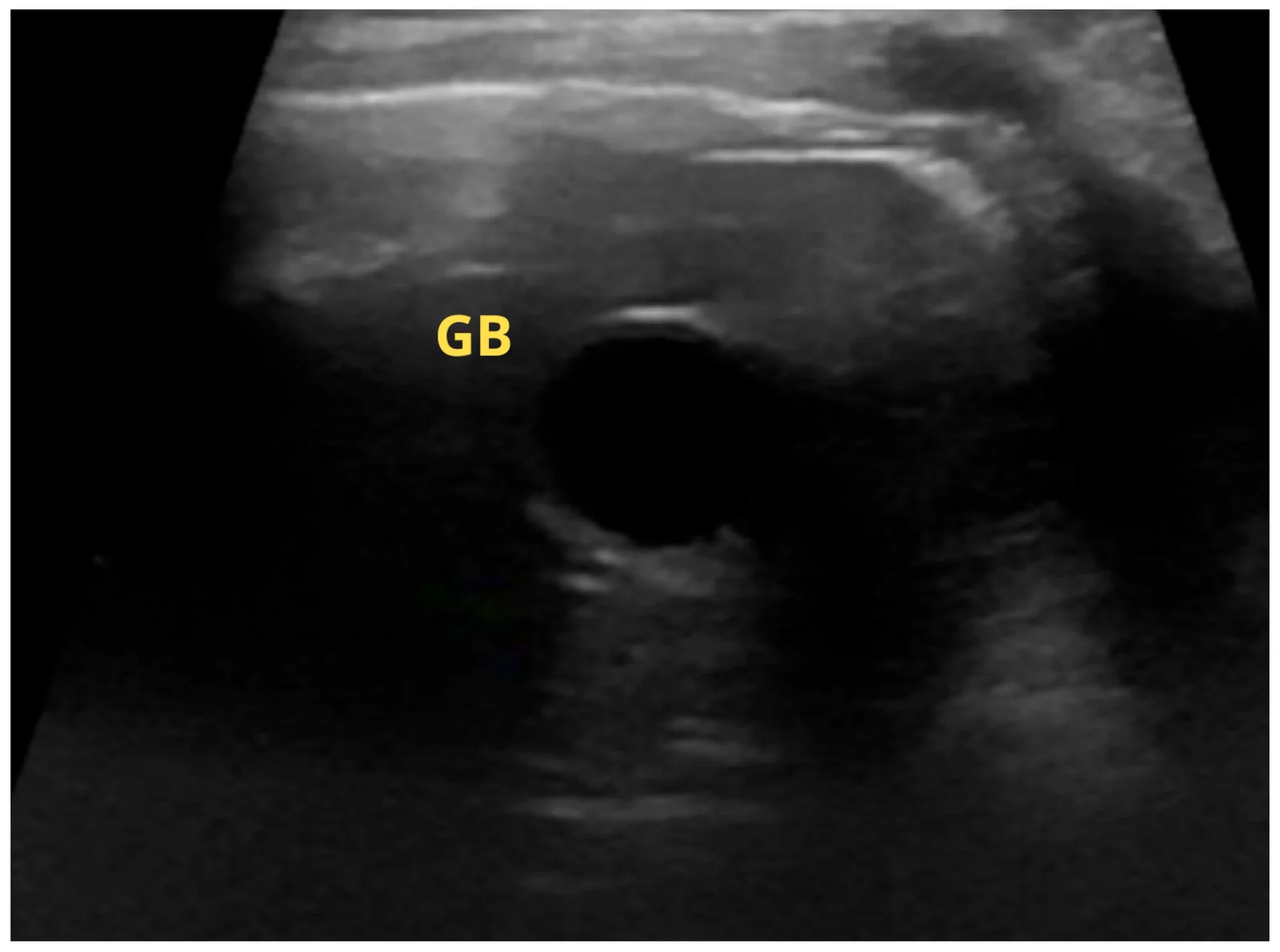
Follow-up abdominal ultrasonography of the gallbladder (GB) in the 8-year-old female Pug (Case 1), 30 days after combined pharmacological and nutritional management. The gallbladder is no longer distended, and intraluminal content is clear with no echogenic sediment or organized mucus, indicating complete resolution of the gallbladder mucocele (GBM).

At the scheduled follow-up, the patient returned for clinical and diagnostic re-evaluation. A thyroid panel (free T4 by dialysis and endogenous TSH) was performed, ruling out hypothyroidism. Follow-up abdominal ultrasonography demonstrated complete resolution of the GBM, with intraluminal content entirely clear and no echogenic sediment or organized mucus observed, indicating restoration of biliary dynamics (Figure 2). The patient also achieved a 10.8% reduction in body weight (from 8.3 kg to 7.4 kg) within the 30-day period. This corresponded to an average weekly weight loss of approximately 2.7%, accompanied by improved activity levels and general vitality, demonstrating a favorable response to the metabolic weight-loss program. The MMS remained at 2/3. Given these results, pharmacological treatment was discontinued, while nutritional management was maintained to support continued improvement in body condition.

### 2.2. Case 2

The second patient, a six-year-old male mixed-breed dog weighing 6.0 kg, was referred for endocrine evaluation following the incidental ultrasonographic detection of mild unilateral adrenal hyperplasia. The clinical history included recurrent calcium oxalate urolithiasis and recent episodes of polyphagia, although no overt signs suggestive of hypercortisolism were observed. Upon physical examination, the patient presented with stable vital parameters but was diagnosed with clinical obesity, with a BCS of 8/9 [16]. A mild discrepancy between adiposity and lean mass was noted, as the dog exhibited mild muscle wasting with an MMS of 2/3 [17]. To investigate suspected adrenal involvement, an adrenocorticotropic hormone (ACTH) stimulation test was performed.

**Figure 3:**
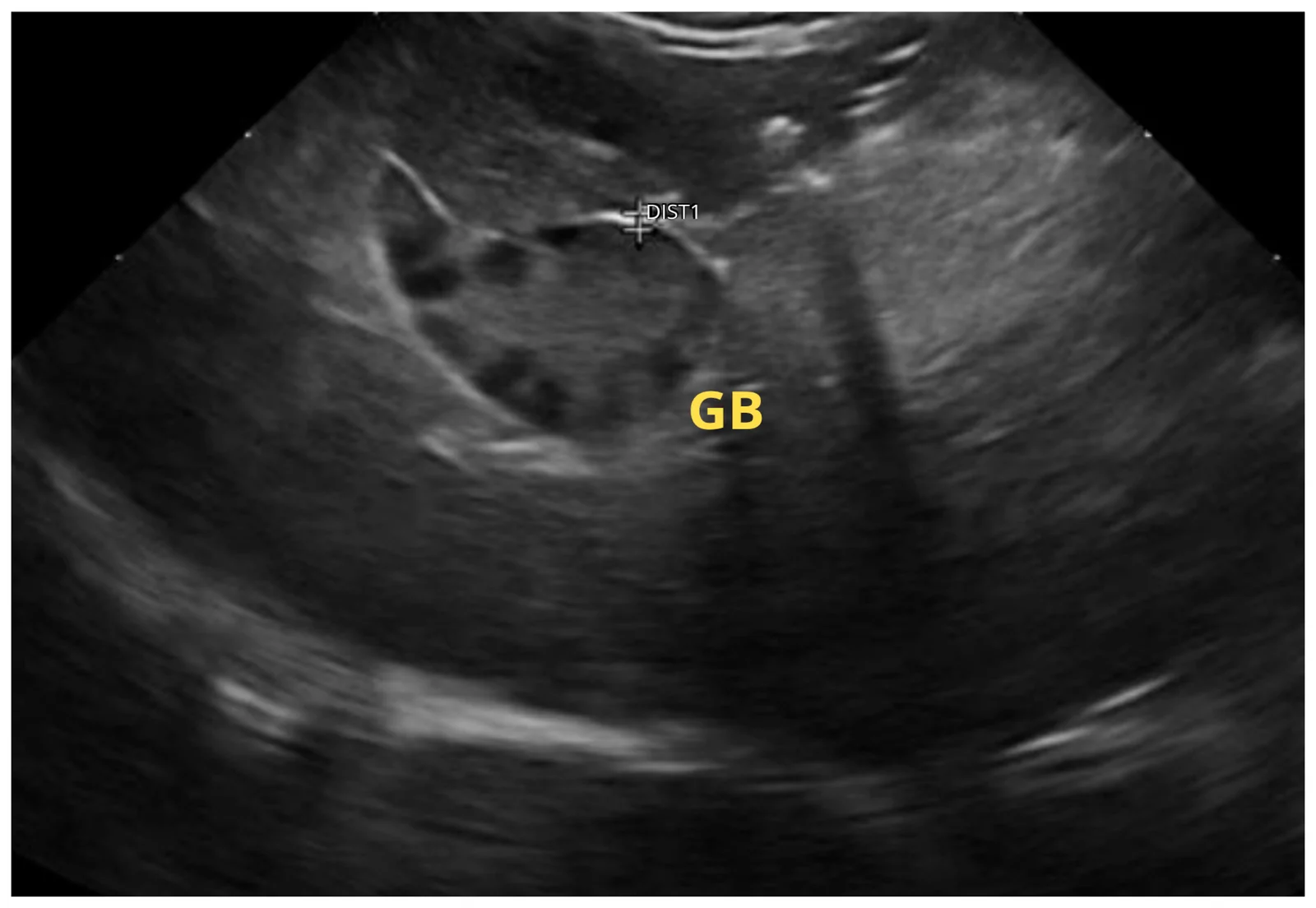
Abdominal ultrasonography of the gallbladder (GB) in the six-year-old male mixed-breed dog (Case 2). The gallbladder is distended, containing immobile, hyperechoic material with a striated pattern, consistent with a gallbladder mucocele (GBM).

During a subsequent follow-up for urolithiasis at the internal medicine service, a comprehensive serum biochemical panel revealed marked elevations in ALP activity (1572 U/L) and significant dyslipidemia, including hypercholesterolemia (343 mg/dL; 125–270 mg/dL) and hypertriglyceridemia (202 mg/dL). Concurrent abdominal ultrasonography confirmed the presence of a bladder stone and unilateral adrenal hyperplasia. Importantly, the imaging also revealed a distended gallbladder containing immobile, hyperechoic material with a striated pattern, consistent with a GBM (Figure 3).

The ACTH stimulation test ruled out hypercortisolism, indicating that the adrenal hyperplasia was non-functional. Given the incidental detection of GBM in an asymptomatic patient, conservative clinical management was prioritized. The pharmacological regimen mirrored that of Case 1. Concurrently, a commercial weight-loss diet was prescribed. The patient’s target body weight was established at 4.8 kg, corresponding to a 20% reduction [14] from the initial body weight (6.0 kg). The metabolic energy was determined using the following equation: ERWL = 70 × (ideal body weight)^0.75^. Clinical and ultrasonographic reassessment was scheduled 30 days after initiation of the combined pharmacological and nutritional protocol.

**Figure 4:**
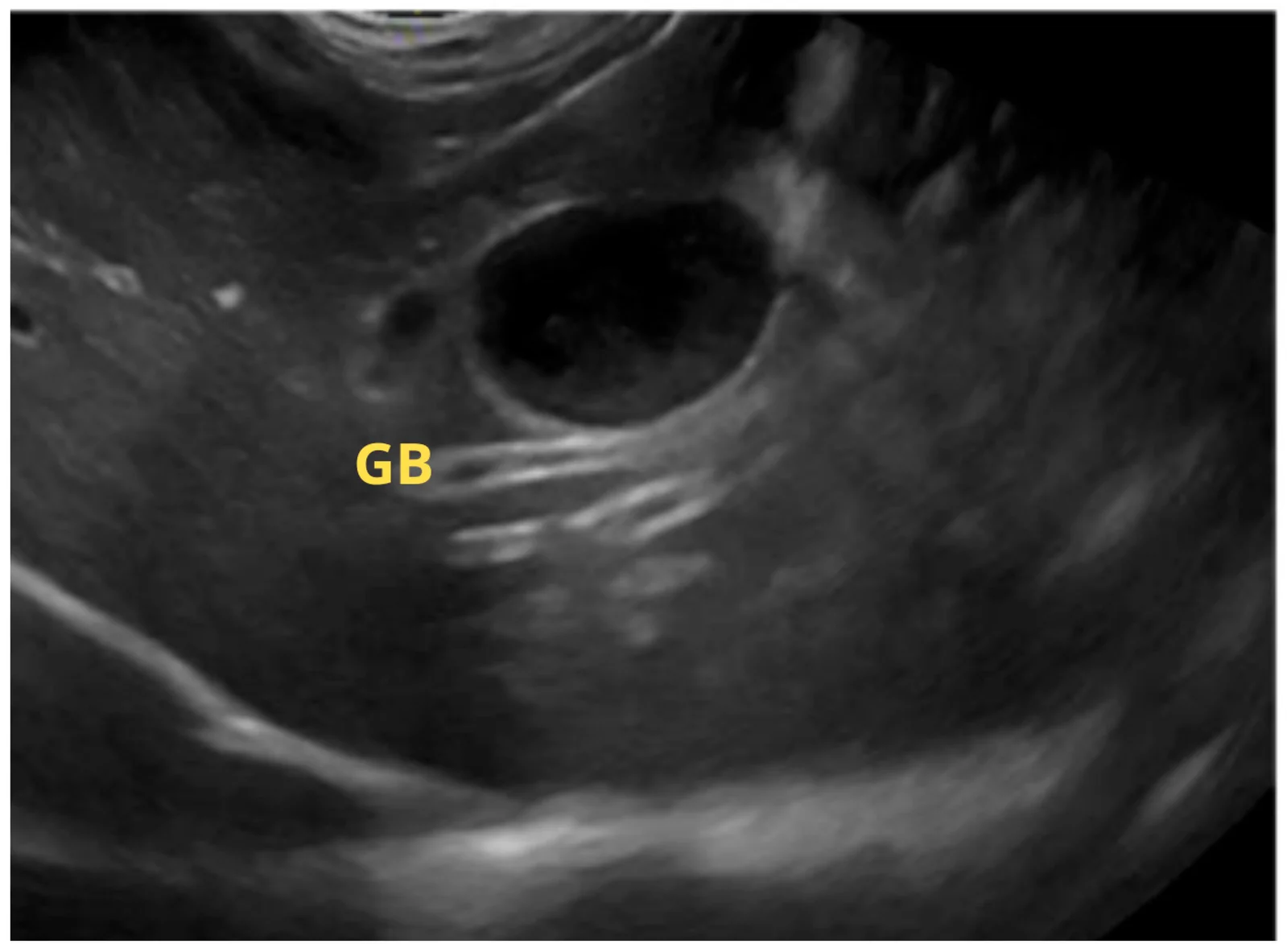
Abdominal ultrasonography of the gallbladder (GB) in the six-year-old male mixed-breed dog (Case 2) after 60 days of combined pharmacological and nutritional management. The gallbladder shows a reduction in intraluminal echogenic material, with mild anechoic striations, indicating partial resolution of the mucocele.

Although a 30-day reassessment was initially planned, follow-up occurred at 60 days. During this interval, the patient showed marked metabolic and clinical improvement, including a 13.3% reduction in body weight, maintenance of an MMS of 2/3, and a decrease in serum lipids (cholesterol: 208 mg/dL; triglycerides: 170 mg/dL) and ALP (676 U/L). The complete laboratory results for the case are presented in Supplementary Table S2. Follow-up ultrasonography revealed a substantial reduction in intraluminal echogenic material, now displaying mild anechoic striations, consistent with partial resolution of the GBM (Figure 4). The combined pharmacological and nutritional protocol was therefore maintained.

### 2.3. Case 3

The third patient, an 11-year-old male Poodle weighing 13.8 kg, was referred for an endocrinology consultation due to refractory obesity and chronic dermatological issues. Despite the owner’s report of a prolonged hypocaloric dietary trial, the patient remained clinically obese, with a BCS of 8/9, and exhibited mild muscle wasting (MMS 2/3) [16,17]. Dermatological examination revealed generalized desquamative and hyperkeratotic lesions, which were under concurrent management by a veterinary dermatologist with topical therapy. Treatment included a therapeutic shampoo based on miconazole and chlorhexidine, as well as moisturizing cream based on ceramides, silicone, and macadamia oil. Otological treatment consisted of gentamicin (0.3%) and miconazole (1%). Initial laboratory screening identified normocytic normochromic anemia, along with marked hypertriglyceridemia (241.6 mg/dL) and hypercholesterolemia (450.1 mg/dL); full laboratory results are presented in Supplementary Table S3.

**Figure 5:**
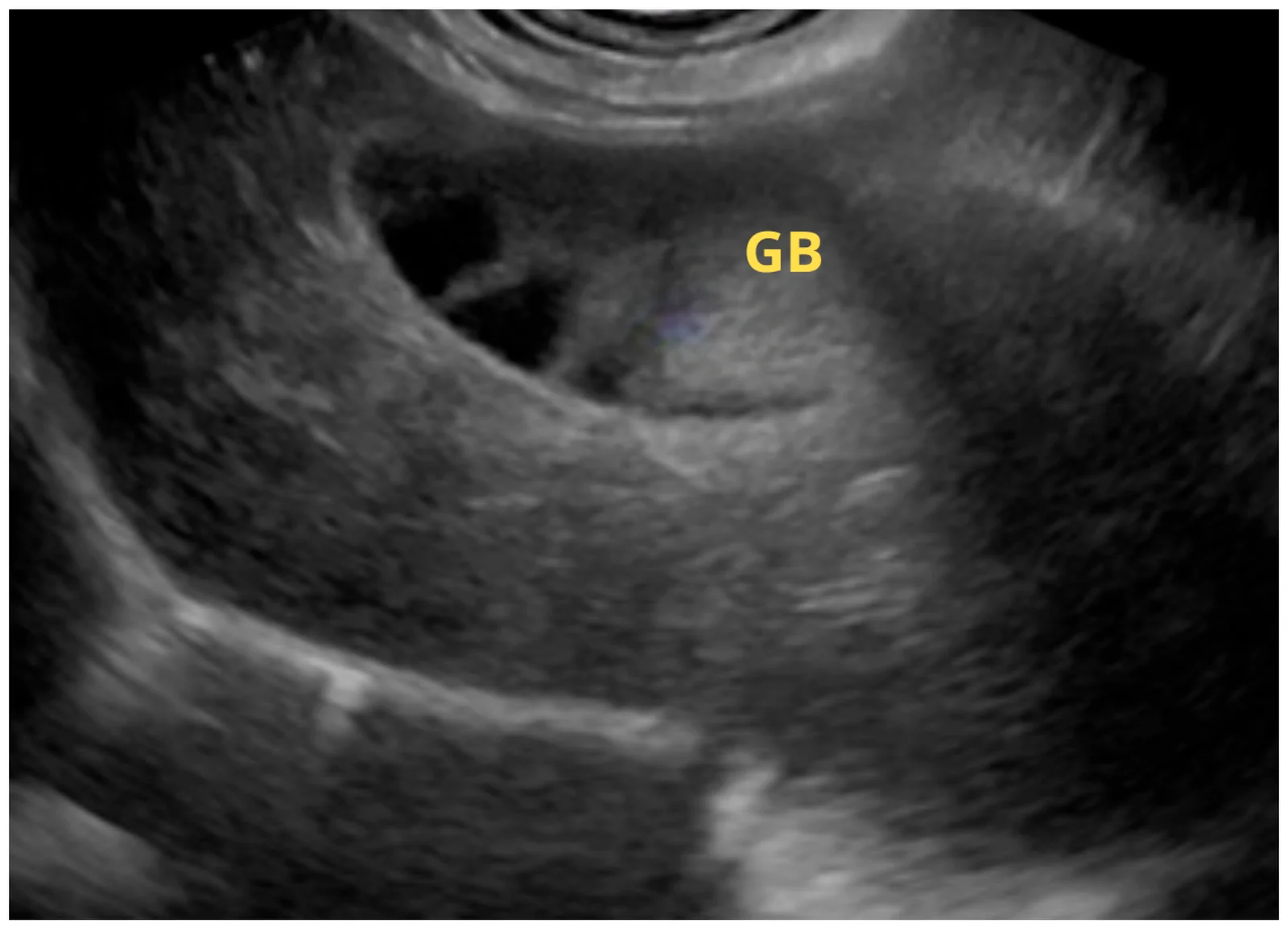
Abdominal ultrasonography of the gallbladder (GB) in the 11-year-old male Poodle (Case 3). The gallbladder is distended and contains immobile, hyperechoic material with a striated pattern radiating from the center to the periphery, producing a characteristic “kiwi fruit” appearance consistent with gallbladder mucocele (GBM).

Given the suspicion of underlying metabolic dysfunction, a comprehensive diagnostic workup was initiated. Thyroid testing confirmed primary hypothyroidism, with low free T4 by dialysis (0.21 ng/dL; reference: 0.82–3.65 ng/dL) and normal TSH at 0.25 ng/dL (reference: 0.10–0.60 ng/dL). Abdominal ultrasonography revealed a distended gallbladder containing immobile, hyperechoic material with a striated pattern radiating from the center to the periphery, producing a characteristic “kiwi fruit” appearance consistent with GBM (Figure 5). The final diagnoses included primary hypothyroidism, secondary hyperlipidemia, obesity, and GBM.

Considering the absence of acute biliary symptoms and the owner’s decision to decline surgical intervention, a conservative clinical approach was initiated. Therapeutic management aimed to both resolve biliary stasis and stabilize the underlying endocrine dysfunction. Hormone replacement therapy for hypothyroidism started with levothyroxine (21 µg/kg, once daily). The pharmacological protocol for GBM was maintained following the same framework applied in previous cases. For nutritional management, the patient was transitioned to the same commercial weight-loss diet used in Case 2.

**Figure 6:**
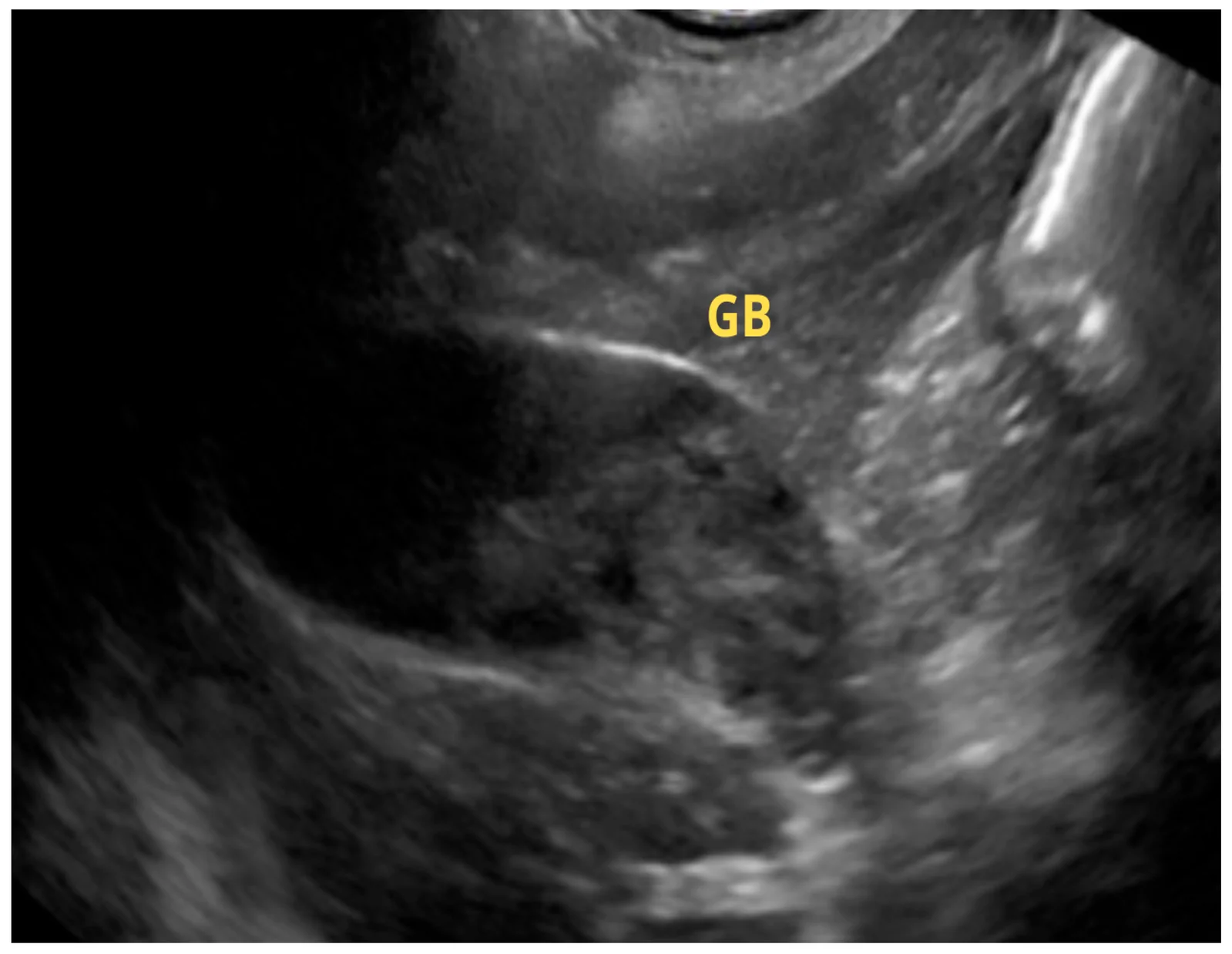
Abdominal ultrasonography of the gallbladder (GB) in the 11-year-old male Poodle (Case 3) after 30 days of combined clinical and nutritional management. The gallbladder shows reduced intraluminal echogenicity with the appearance of mild anechoic areas, consistent with decreased content density and partial sonographic resolution of gallbladder mucocele (GBM).

At the 30-day reassessment, despite the owner not having completed the requested thyroid monitoring or follow-up blood chemistry, objective clinical progress was evident. The patient achieved a 4.3% reduction in body weight and maintenance of an MMS of 2/3, reflecting a positive response to the combined metabolic stimulation of pharmacological and dietary interventions. Follow-up abdominal ultrasonography demonstrated notable improvement in gallbladder structure; the previously dense, striated intraluminal content now showed reduced echogenicity with the appearance of discrete anechoic areas, consistent with decreased content density and partial sonographic resolution of GBM (Figure 6). Given this favorable ultrasonographic evolution and the owner’s continued preference for non-invasive management, the combined clinical and nutritional approach was maintained.

### 2.4. Case 4

**Figure 7:**
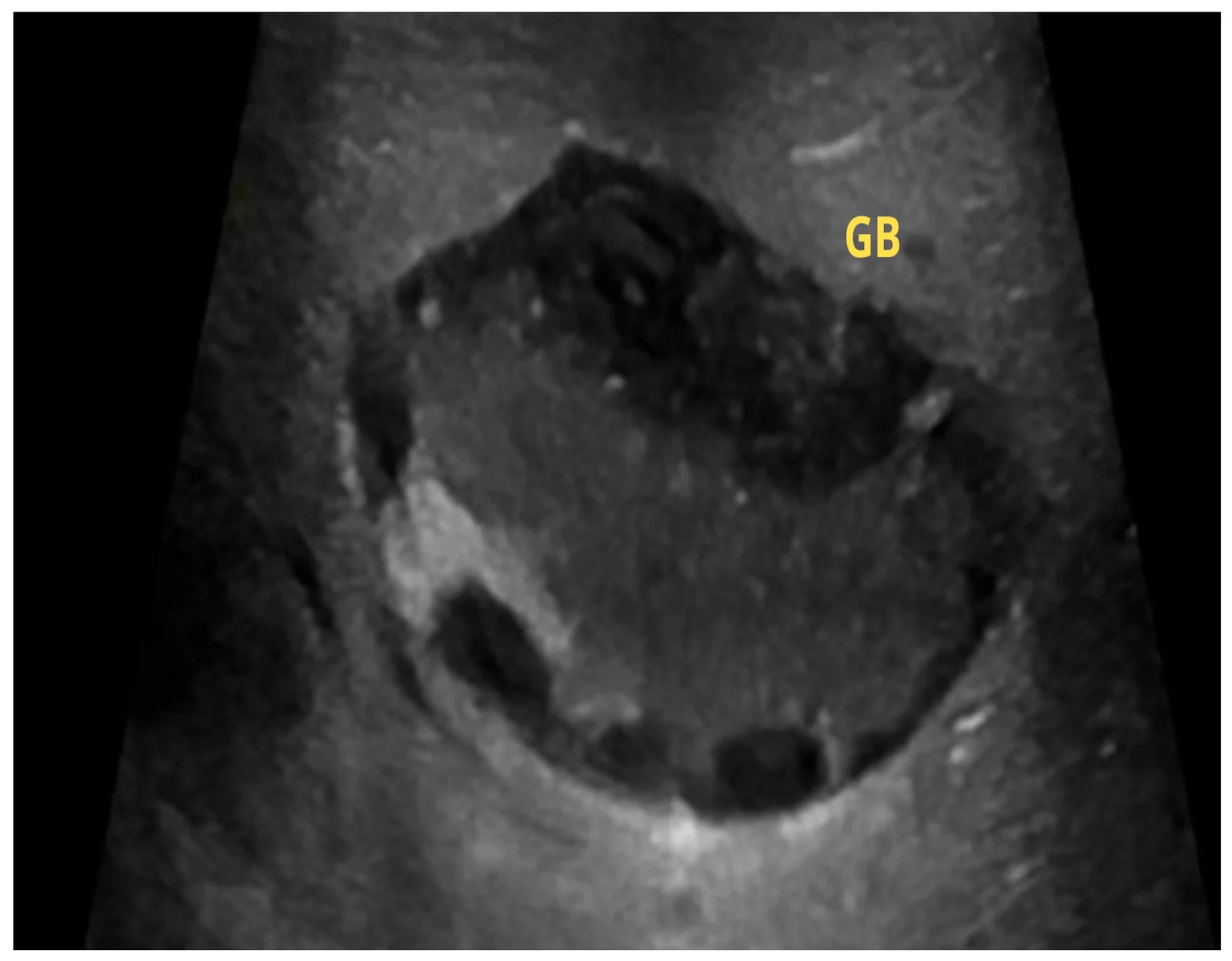
Abdominal ultrasonography of the gallbladder (GB) in the 13-year-old male Poodle (Case 4). The gallbladder is distended and contains immobile, amorphous, hyperechoic material with a striated pattern extending from the center to the periphery, consistent with a gallbladder mucocele (GBM).

The fourth patient, a 13-year-old male Poodle weighing 4.8 kg, was referred after incidental detection of a GBM during routine geriatric ultrasonographic screening. The patient was clinically asymptomatic, with no prior history of biliary or gastrointestinal disorders. Physical examination revealed a bright, alert, and responsive dog with no significant abnormalities. Despite an ideal body condition score (BCS 4/9) [16], mild muscle wasting was noted (MMS 2/3) [17], consistent with early-stage senile sarcopenia. Abdominal ultrasonography showed a distended gallbladder containing immobile, amorphous, hyperechoic material with a striated pattern radiating from the center to the periphery, characteristic of a GBM (Figure 7).

Laboratory evaluation revealed hypertriglyceridemia (240.7 mg/dL) as the only metabolic abnormality. Based on the diagnosis of GBM and concurrent hyperlipidemia, a conservative therapeutic plan was initiated. The pharmacological protocol followed the same regimen applied in previous cases, while the nutritional strategy was tailored to the patient’s ideal body condition. Instead of caloric restriction, a maintenance low-fat diet was prescribed. The daily MER was calculated according to the guidelines of the FEDIAF (2025) [15], using the equation: MER = 95 × (ideal body weight)^0.75^ for inactive adult dogs. Based on the patient’s body weight (4.8 kg), the estimated MER was 307.8 kcal/day.

**Figure 8:**
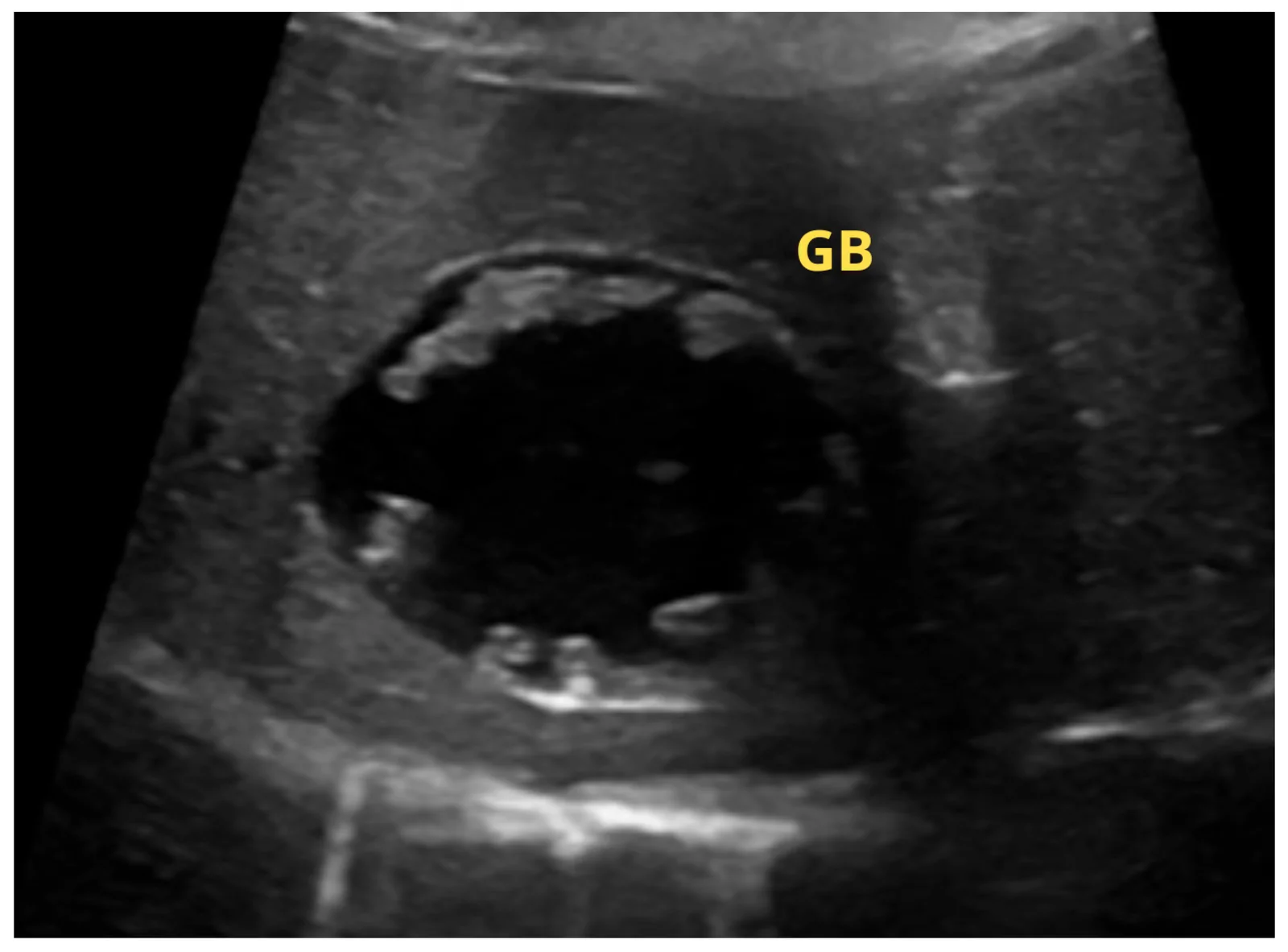
Abdominal ultrasonography of the gallbladder (GB) in the 13-year-old male Poodle (Case 4) after 90 days of clinical and nutritional management. The gallbladder shows a reduction in the organized striated pattern, with the remaining contents appearing as echogenic biliary sludge, indicating resolution of the characteristic ultrasonographic features of the gallbladder mucocele (GBM) with residual biliary sludge.

At the 60-day reassessment, ultrasonography showed no significant change, with the gallbladder still containing a large volume of immobile, striated amorphous material. Given the absence of clinical deterioration and the owner’s financial constraints precluding surgical intervention, medical management was maintained. At the 90-day follow-up, however, ultrasonography demonstrated a marked reduction in the organized striated pattern, with the gallbladder contents appearing as simple echogenic biliary sludge (Figure 8). This improvement was accompanied by normalization of serum triglyceride levels (158.7 mg/dL). Complete laboratory results for this case are presented in Supplementary Table S4, and the combined pharmacological and nutritional protocol was continued.

## 3. Discussion

This case series, with concise case summaries provided in Supplementary Table S5, suggests that clinical and ultrasonographic improvement may be observed in dogs with GBM managed with a combined pharmacological and nutritional approach, particularly in patients with underlying metabolic alterations and in those not considered ideal candidates for surgical intervention. Across all cases, improvements were observed in parallel with metabolic control, especially in relation to lipid profiles. These findings offer insights into the potential role of individualized nutritional management as an adjunctive component of a multimodal conservative approach to this condition.

Gallbladder mucocele is more frequently reported in older dogs and is often identified incidentally during imaging performed for unrelated conditions [4,18,19]. This pattern was also observed in the present case series, in which three out of four patients were senior and asymptomatic at the time of diagnosis. The absence of overt clinical signs is consistent with the natural course of GBM and reinforces the importance of early detection before progression to severe complications [20,21].

In the present case series, conservative management was selected because the owners declined surgical intervention in all four cases. This decision was primarily influenced by the advanced age of the patients and the absence of clinical signs or evidence of gallbladder rupture, supporting the decision to initially pursue medical management. Therefore, conservative management may represent a viable alternative for clinically stable and asymptomatic dogs when surgery is declined or not considered appropriate [22].

Another consistent finding across all cases was the presence of metabolic alterations, particularly hyperlipidemia, which was observed in all patients, along with overweight or obesity in three cases and hypothyroidism in one. These findings aligned with previous reports identifying metabolic dysfunction as a major predisposing factor for GBM [23,24]. Hyperlipidemia has been associated with impaired gallbladder motility and altered bile composition, contributing to biliary stasis and mucocele formation [25]. Given its presence across all cases, lipid metabolism appears to play an important role not only in the development but also in the progression of GBM [26,27]. In the present series, ultrasonographic improvement occurred concurrently with reductions in circulating lipid concentrations, supporting the relevance of targeting metabolic dysfunction as part of the therapeutic strategy.

Overweight and obesity were identified in three of the four patients and may have further contributed to the metabolic disturbances associated with GBM. Obesity is recognized as a state of chronic low-grade inflammation and has been associated with alterations in lipid metabolism [28], insulin sensitivity [29], and biliary function [30], all of which may favor gallbladder disease [1,30]. While weight control was not the sole therapeutic intervention implemented in the present cases, patients managed under structured weight-loss programs exhibited concurrent improvements in body condition, lipid profiles, and ultrasonographic findings, suggesting an association between improvement in obesity-associated metabolic dysfunction and the favorable clinical response observed.

Although identified in only one patient in the present series, hypothyroidism has been reported in dogs with GBM with a prevalence ranging from 5% to 14% [31]. In the affected patient, the inclusion of hormone replacement therapy alongside the combined pharmacological and nutritional management was accompanied by progressive clinical and ultrasonographic improvement. However, the individual contribution of levothyroxine cannot be determined because thyroid function and follow-up biochemical parameters were not reassessed at the time of re-evaluation. Nevertheless, improvement in the underlying endocrine disorder may have contributed to the overall response.

Given the multifactorial etiology of GBM and the metabolic alterations identified in the present cases, a multimodal therapeutic approach was adopted. Medical management included UDCA, which has been used to support bile flow and reduce the accumulation of cytotoxic bile acids [32]. SAMe and silymarin were also administered because of their potential hepatoprotective effects and their use in supporting biliary function [22]. This therapeutic strategy has demonstrated reductions in cholestatic and hepatic injury markers, while also improving ultrasonographic parameters such as gallbladder sludge accumulation and hepatic echogenicity in dogs with subclinical GBM [22].

Given the consistent presence of hyperlipidemia in the present series, lipid-lowering therapy was also incorporated, including bezafibrate. Although these pharmacological interventions may have contributed to the clinical improvement observed, previous studies suggest that medical management alone may not consistently promote complete GBM resolution when underlying metabolic disturbances remain uncontrolled [20].

In this context, nutrition should be considered a key component in the clinical management of GBM, particularly given the metabolic nature of the disease [33,34,35]. Low-fat diets are traditionally recommended due to their effects on bile composition and gallbladder responsiveness to cholecystokinin (CCK) [10]. However, because dietary modification was implemented concurrently with pharmacological therapy and, in some patients, structured weight-loss programs, the individual contribution of nutrition to the observed clinical response cannot be determined. Nevertheless, these cases suggest that nutritional management may have supported the overall therapeutic strategy by contributing to the control of metabolic disturbances commonly associated with GBM.

In this series, Case 1 received a diet with a higher fat density (22.4 g/1000 kcal) than Case 4 (14.6 g/1000 kcal). Nevertheless, based on fat intake per kg of metabolic body weight, fat intake remained relatively similar between cases (1.57 vs. 1.39 g/kg^0.75^, respectively). Importantly, Case 1 underwent a structured weight-loss program with caloric restriction, whereas Case 4 received maintenance energy intake. Despite the slightly higher fat intake, Case 1 exhibited faster ultrasonographic improvement. Although the individual contribution of dietary composition cannot be distinguished from that of caloric restriction and weight loss, these findings suggest that improvement in obesity-associated metabolic dysfunction may have contributed to the observed clinical response. Similar findings were observed in Cases 2 and 3, in which weight reduction occurred concurrently with improvements in lipid profiles (Case 2) and gallbladder ultrasonographic patterns.

Omega-3 fatty acids were administered at a dose equivalent to 120 mg of EPA and DHA/kg BW^0.75^ [36]. These fatty acids have well-established triglyceride-lowering and anti-inflammatory effects in dogs and may have contributed to the improvement in lipid metabolism observed throughout the follow-up period [37]. Although their individual contribution cannot be determined in the present cases, omega-3 supplementation was incorporated as part of the overall nutritional strategy aimed at controlling hyperlipidemia. Together, these findings reinforce the importance of integrated dietary approaches targeting lipid metabolism as part of GBM management.

Beyond fat restriction, the dietary strategies adopted in the present series also differed in carbohydrate, protein, and fiber composition. Cases 2 and 3 received diets containing complex carbohydrate sources and higher total dietary fiber content, whereas soluble fibers, including psyllium and beet pulp, may contribute to lipid modulation and cholesterol homeostasis through their effects on intestinal lipid absorption and bile acid metabolism [38,39]. It is worth noting that Case 2 showed progressive reductions in circulating lipid concentrations, while Case 2 and 3 showed improvements in ultrasonographic findings while consuming high-fiber diets. However, the interpretation of these observations remains limited by the lack of information regarding the proportion of soluble and insoluble fiber in diets, as well as by differences in fiber characterization among products (crude fiber versus total dietary fiber). Therefore, the specific contribution of dietary fiber to the clinical outcomes observed in the present cases cannot be determined.

The protein intake also differed among cases; however, all dogs received amounts exceeding the minimum recommended allowance for adult dogs according to FEDIAF (2025) [15]. Although specific protein requirements for dogs with GBM have not been established, maintaining adequate protein intake is important to preserve lean body mass, particularly in patients undergoing weight-loss programs or presenting concurrent metabolic disorders.

The cases presented here illustrate that the combined use of pharmacological therapy and individualized nutritional strategies may contribute to the clinical management of GBM, as evidenced by improvements in ultrasonographic patterns and metabolic parameters. However, the rate of clinical response varied among patients, likely reflecting differences in metabolic status, body condition, underlying comorbidities, and dietary management. In particular, patients undergoing structured weight-loss protocols exhibited more rapid metabolic and ultrasonographic improvement, suggesting that correction of obesity-associated metabolic dysfunction may positively influence biliary recovery. However, the contribution of weight loss cannot be separated from that of the other concurrent interventions.

Overall, the present study did not aim to establish a single standard of care for GBM, and its findings provide preliminary observations on the use of a multimodal and individualized therapeutic approach. The favorable outcomes observed across patients with distinct predisposing factors suggest that combined medical and nutritional management may represent a complementary or alternative strategy in selected asymptomatic or clinically stable dogs.

## 4. Conclusions

Medical management of GBM combined with targeted nutritional strategies may represent a potential approach in selected asymptomatic patients, particularly those not eligible for surgery or diagnosed in the early, uncomplicated stages of the disease.

## Figures and Tables

**Table 1:** Nutritional composition (per 1000 kcal of metabolizable energy) and main ingredients of the diets consumed by dogs before the diagnosis of gallbladder mucocele.

Nutrient	Patient 1 and 4 *	Patient 2 **	Patient 3 ***
Crude protein, g/1000 kcal	47.1	47.8	100.0
Ether extract, g/1000 kcal	42.1	46.6	28.3
Mineral matter, g/1000 kcal	22.8	13.5	30.0
Crude fiber, g/1000 kcal ^†^	7.7	3.7	43.3
Nitrogen-free extract, g/1000 kcal	99.6	111.5	101.7
Metabolizable energy, kcal/kg	4038	4080	3000

Legend: GI = gastrointestinal; ME = metabolizable energy. ^†^ fiber values obtained from available dietary information. * commercial hydrolyzed diet main ingredients: brewers’ rice, hydrolyzed soy protein, poultry fat, pork fat, beet pulp, soybean oil, fish oil, borage oil, fructooligosaccharides (FOS), DL-methionine, taurine and poultry liver flavor. ** commercial urinary diet main ingredients: poultry by-product meal, brewers’ rice, oat groats, potato starch, egg powder, pork crackling meal, poultry fat, flaxseed, powdered cellulose, fish oil, mannanoligosaccharides (MOS), DL-methionine, taurine and L-carnitine. *** commercial weight-loss diet main ingredients: poultry, poultry by-product meal, cassava starch, cassava flour, sugarcane fiber, beet pulp, guava fiber, apple, poultry fat, fish oil, flaxseed meal, psyllium, fructooligosaccharides (FOS), inulin, probiotics, hydrolyzed collagen, glucosamine, chondroitin sulfate, DL-methionine, taurine and L-carnitine.

**Table 2:** Nutritional composition (per 1000 kcal of metabolizable energy) and main ingredients of the diets prescribed for dogs with gallbladder mucocele.

Nutrient	Homemade Diet (Case 1) *	Commercial Weight Loss (Cases 2 & 3) **	Commercial Low-Fat GI (Case 4) ***
Crude protein, g/1000 kcal	124.7	108.0	58.2
Ether extract, g/1000 kcal	22.4	27.4	14.6
Mineral matter, g/1000 kcal	8.0	22.8	23.9
Crude fiber, g/1000 kcal ^†^	-	45.6	12.5
Total dietary fiber, g/1000 ^†^ kcal	16.3	59.3	-
Carbohydrates, g/1000 kcal	55.4	-	-
Nitrogen-free extract, g/1000 kcal	-	70.0	148.5
Metabolizable energy, kcal/kg	4410	3286	3434

Legend: GI = gastrointestinal; ME = metabolizable energy. ^†^ fiber measurements were reported as crude fiber or total dietary fiber according to the available information for each diet and should not be considered directly comparable. * homemade weight-loss diet main ingredients: pork loin, sweet potato, green beans, chayote squash and salt. Formulated based on individual metabolic requirements using specialized formulation software (Diet Lab). ** commercial weight-loss diet main ingredients: poultry byproduct meal, pork crackling meal, wheat gluten, egg powder, dehydrated swine blood plasma, ground peas, barley, brewers’ rice, cellulose, sugar cane fiber, beet pulp, chicken fat, fish oil, *Acacia nilotica*, and turmeric. *** commercial low-fat gastrointestinal diet main ingredients: poultry byproduct meal, pork fat, fish oil, brewers’ rice, oat groats, ground corn, beet pulp, soy fiber, corn gluten meal, psyllium husk, and yeast products.

**Table 3:** Daily energy and nutrient intake expressed per kilogram of metabolic body weight (BW^0.75^) in dogs with gallbladder mucocele.

Variables	Patient 1	Patient 2	Patient 3	Patient 4
Body weight, kg	7.05 ^‡^	4.80 ^‡^	13.80	4.80
Body condition score ^1^	7/9	8/9	8/9	4/9
Metabolic body weight, kg^0.75^	4.32 ^†^	3.24 ^†^	7.15 ^††^	3.24 ^††^
Daily energy intake, kcal/day	302.4 *	226.8 **	679.3 **	307.8 **
Daily food intake, g/day	68.57	69.00	206.70	89.60
Energy, kcal/kg BW^0.75^/day	70.0	70.00	95.0	95.0
Protein, g/kg BW^0.75^/day	8.73	7.56	10.26	5.53
Fat, g/kg BW^0.75^/day	1.57	1.92	2.60	1.39
TDF, g/kg BW^0.75^/day	1.14	4.15	5.63	-

Legend: BW = body weight; TDF = total dietary fiber. ^‡^ ideal body weight, defined as 15–20% below the initial body weight. ^†^ calculated using (ideal body weight)^0.75^. ^††^ calculated using (body weight)^0.75^. * calculated using the energy requirement for weight loss (ERWL) equation according to Brooks et al. (2014): 70 × (ideal body weight)^0.75^ [14]. ** calculated using the maintenance energy requirement (MER) for inactive dogs according to FEDIAF (2025): 95 × (body weight)^0.75^ [15]. ^1^ assessment of body condition score according to Laflamme (1997) [16]. TDF data were unavailable for the commercial low-fat gastrointestinal diet.

## Data Availability

The data presented in this study are available from the corresponding author upon reasonable request. The data are not publicly available due to patient privacy and confidentiality considerations.

## Supplementary Materials

The following supporting information can be downloaded at: https://www.mdpi.com/article/10.3390/ani16172771/s1, Table S1. Complete laboratory findings of Case 1; Table S2. Complete laboratory findings of Case 2 (Initial evaluation/Follow-up); Table S3. Complete laboratory findings of Case 3; Table S4. Complete laboratory findings of Case 4 (Initial evaluation/Follow-up); Table S5: Summary of the main clinical, metabolic findings, ultrasonographic, nutritional, therapeutic, and follow-up findings in four dogs with gallbladder mucocele (GBM).

## Abbreviations

The following abbreviations are used in this manuscript:

ALP	Alkaline phosphatase
ALT	Alanine aminotransferase
BCS	Body condition score
BW	Body weight
CCK	Cholecystokinin
ERWL	Energy requirement for weight loss
GB	Gallbladder
GBM	Gallbladder mucocele
GI	Gastrointestinal
LDDS	Low-dose dexamethasone suppression
ME	Metabolizable energy
MER	Maintenance energy requirement
MMS	Muscle mass score
SAMe	S-adenosylmethionine
T4	Thyroxine
TDF	Total dietary fiber
TSH	Thyroid-stimulating hormone
TW	Target body weight
UDCA	Ursodeoxycholic acid
