# Supplementary material for: Clinical and Nutritional Management of Gallbladder Mucocele in Dogs: A Four-Case Series
Source: Animals (Basel). 2026 Sep 3;16(17):2771. doi: 10.3390/ani16172771 (PMC13565239; doi:10.3390/ani16172771)
Supplement: Supplementary file 1 [file animals-16-02771-s001.zip › animals-4480302-supplementary.pdf]

Table S1. Complete laboratory findings of Case 1.

Laboratory panel	Parameter	Result	Reference interval
Complete blood count	Erythrocytes (M/µL)	6.13	5.65 - 8.87
	Hematocrit (%)	39.50	37.3 - 61.7
	Hemoglobin (g/dL)	14.10	13.1 - 20.5
	MCV (fL)	64.40	61.6 - 73.5
	MCH (pg)	23.0	21.2 - 25.9
	MCHC (g/dL)	35.7	32.0 - 37.9
	RDW (%)	15.8	13.6 - 21.7
	Reticulocytes (K/µL)	65.5	10.0 - 110.0
	Leukocytes (K/µL)	16.00	5.05 - 16.76
	Neutrophils (K/µL)	10.50	2.95 - 11.64
	Lymphocytes (K/µL)	4.50	1.05 - 5.10
	Monocytes (K/µL)	0.87	0.16 - 1.12
	Eosinophils (K/µL)	0.16	0.06 - 1.23
	Basophils (K/µL)	0.03	0.00 - 0.10
	Platelets (K/µL)	250	148 - 484
Serum biochemistry	Glucose (mg/dL)	103	74 - 143
	Creatinine (mg/dL)	0.9	0.5 - 1.8
	Urea (mg/dL)	25	7 - 27
	Total proteins (g/dL)	6.7	5.2 – 8.2
	Albumin (g/dL)	2.6	2.3 – 4.0
	ALT (U/L)	69	10 - 125
	ALP (U/L)	468	23 - 212
	Cholesterol (mg/dL)	252	125 - 270
	Triglycerides (mg/dL)	324	50 - 100
Endocrine analyses	TSH (ng/dL)	0.44	0.10 - 0.60
	Free T4 (equilibrium dialysis) (ng/dL)	2.65	0.82 - 3.65

Note: Post-treatment laboratory data were unavailable for Case 1; therefore, only baseline laboratory findings are presented. Reference intervals correspond to those provided by the clinical pathology laboratory where the analyses were performed. Legend: ALT = alanine aminotransferase; ALP = alkaline phosphatase; TSH = thyroid-stimulating hormone; T4 = thyroxine.

Table S2. Complete laboratory findings of Case 2 (Initial evaluation/ Follow-up).

Laboratory panel	Parameter	Initial evaluation	Follow-up	Reference interval
Complete blood count	Erythrocytes (M/μL)	7.51	-	5.65 - 8.87
	Hematocrit (%)	54.6	-	37.3 - 61.7
	Hemoglobin (g/dL)	20.0	-	13.1 - 20.5
	MCV (fL)	72.7	-	61.6 - 73.5
	MCH (pg)	23.5	-	21.2 - 25.9
	MCHC (g/dL)	36.7	-	32.0 - 37.9
	RDW (%)	15.4	-	13.6 - 21.7
	Reticulocytes (K/μL)	65.5	-	10.0 - 110.0
	Leukocytes (K/μL)	14.35	-	5.05 - 16.76
	Neutrophils (K/μL)	6.97	-	2.95 - 11.64
	Lymphocytes (K/μL)	4.69	-	1.05 - 5.10
	Monocytes (K/μL)	0.90	-	0.16 - 1.12
	Eosinophils (K/μL)	0.41	-	0.06 - 1.23
	Basophils (K/μL)	0.00	-	0.00 - 0.10
	Platelets (K/μL)	199	-	148 - 484
Serum biochemistry	Glucose (mg/dL)	120	-	74 - 143
	Creatinine (mg/dL)	0.7	-	0.5 - 1.8
	Urea (mg/dL)	15	-	7 - 27
	Total proteins (g/dL)	7.7	-	5.2 – 8.2
	Albumin (g/dL)	3.2	-	2.3 – 4.0
	ALT (U/L)	73	-	10 - 125
	ALP (U/L)	1572	676	23 - 212
	Cholesterol (mg/dL)	343	208	125 - 270
	Triglycerides (mg/dL)	202	170	50 - 100
Endocrine analysis	Basal cortisol (μg/dL)	2.36	-	1.0 - 4.60
	Post-ACTH cortisol (μg/dL)	8.84	-	5.0 - 17.00

Note: Follow-up laboratory evaluation was not performed for all parameters; only available post-treatment results are reported. Unavailable measurements are indicated by "-". Reference intervals correspond to those provided by the clinical pathology laboratory where the analyses were performed. Legend: ALT = alanine aminotransferase; ALP = alkaline phosphatase; ACTH = adrenocorticotropic hormone.

Table S3. Complete laboratory findings of Case 3.

Laboratory panel	Parameter	Result	Reference interval
Complete blood count	Erythrocytes (M/μL)	4.81	5.65 - 8.87
	Hematocrit (%)	35.2	37.3 - 61.7
	Hemoglobin (g/dL)	11.5	13.1 - 20.5
	MCV (fL)	73.2	61.6 - 73.5
	MCH (pg)	23.9	21.2 - 25.9
	MCHC (g/dL)	32.7	32.0 - 37.9
	RDW (%)	15.1	13.6 - 21.7
	Reticulocytes (K/μL)	63.5	10.0 - 110.0
	Leukocytes (K/μL)	10.92	5.05 - 16.76
	Neutrophils (K/μL)	9.63	2.95 - 11.64
	Lymphocytes (K/μL)	2.64	1.05 - 5.10
	Monocytes (K/μL)	0.54	0.16 - 1.12
	Eosinophils (K/μL)	0.07	0.06 - 1.23
	Basophils (K/μL)	0.00	0.00 - 0.10
	Platelets (K/μL)	442	148 - 484
Serum biochemistry	Glucose (mg/dL)	106.00	74 - 143
	Creatinine (mg/dL)	0.7	0.5 - 1.8
	Urea (mg/dL)	8	7 - 27
	Total proteins (g/dL)	7.2	5.2 – 8.2
	Albumin (g/dL)	2.8	2.3 – 4.0
	ALT (U/L)	103	10 - 125
	ALP (U/L)	102	23 - 212
	Cholesterol (mg/dL)	450.1	125 - 270
	Triglycerides (mg/dL)	241.6	50 - 100
Endocrine analyses	TSH (ng/dL)	0.25	0.10 - 0.60
	Free T4 (equilibrium dialysis) (ng/dL)	0.21	0.82 - 3.65

Note: Post-treatment laboratory data were unavailable for Case 3; therefore, only baseline laboratory findings are presented. Reference intervals correspond to those provided by the clinical pathology laboratory where the analyses were performed. Legend: ALT = alanine aminotransferase; ALP = alkaline phosphatase; TSH = thyroid-stimulating hormone; T4 = thyroxine.

Table S4. Complete laboratory findings of Case 4 (Initial evaluation/ Follow-up).

Laboratory panel	Parameter	Initial evaluation	Follow-up	Reference interval
Complete blood count	Erythrocytes (M/μL)	7.50	7.61	5.65 - 8.87
	Hematocrit (%)	49.6	49.2	37.3 - 61.7
	Hemoglobin (g/dL)	17.7	17.9	13.1 - 20.5
	MCV (fL)	66.2	64.7	61.6 - 73.5
	MCH (pg)	23.6	23.5	21.2 - 25.9
	MCHC (g/dL)	35.7	36.3	32.0 - 37.9
	RDW (%)	18.0	19.3	13.6 - 21.7
	Reticulocytes (K/μL)	110.0	65.2	10.0 - 110.0
	Leukocytes (K/μL)	9.54	8.06	5.05 - 16.76
	Neutrophils (K/μL)	7.63	5.83	2.95 - 11.64
	Lymphocytes (K/μL)	1.14	1.16	1.05 - 5.10
	Monocytes (K/μL)	0.53	0.77	0.16 - 1.12
	Eosinophils (K/μL)	0.24	0.62	0.06 - 1.23
	Basophils (K/μL)	0.00	0.01	0.00 - 0.10
	Platelets (K/μL)	289	245	148 - 484
Serum biochemistry	Glucose (mg/dL)	90	-	74 - 143
	Creatinine (mg/dL)	0.6	-	0.5 - 1.8
	Urea (mg/dL)	14	-	7 - 27
	Total proteins (g/dL)	7.1	-	5.2 – 8.2
	Albumin (g/dL)	3.3	-	2.3 – 4.0
	ALT (U/L)	123	-	10 - 125
	ALP (U/L)	210	-	23 - 212
	Cholesterol (mg/dL)	225.6	188.8	125 - 270
	Triglycerides (mg/dL)	240.7	158.7	50 - 100

Note: Follow-up laboratory evaluation was not performed for all parameters; only available post-treatment results are reported. Unavailable measurements are indicated by "-". Reference intervals correspond to those provided by the clinical pathology laboratory where the analyses were performed. Legend: ALT = alanine aminotransferase; ALP = alkaline phosphatase.

Table S5: Summary of the main clinical, metabolic findings, ultrasonographic, nutritional, therapeutic, and follow-up findings in four dogs with gallbladder mucocele (GBM).

Variables	Case 1	Case 2	Case 3	Case 4
**Age/sex/breed**	8-year-old, female, Pug	6-year-old, male, mixed-breed	11-year-old, male, Poodle	13-year-old, male, Poodle
**Initial BW/BCS/MMS**	BW: 8.3 kg<br>BCS: 7/9<br>MMS: 2/3	BW: 6 kg<br>BCS: 8/9<br>MMS: 2/3	BW: 13.8 kg<br>BCS: 8/9<br>MMS: 2/3	BW: 4.8 kg<br>BCS: 4/9<br>MMS: 2/3
**Main clinical/metabolic findings**	Overweight, Hypertriglyceridemia and elevation in ALP	Obesity, hypercholesterolemia, hypertriglyceridemia, elevation in ALP, unilateral adrenal hyperplasia and urolithiasis	Obesity, dermatological alterations, hypercholesterolemia, hypertriglyceridemia, normocytic normochromic anemia and hypothyroidism	Hypertriglyceridemia
**Initial ultrasonographic findings**	Distended GB with immobile, hyperechoic, stellate-patterned content	Distended GB with immobile, hyperechoic, striated-patterned content	Distended GB with immobile, hyperechoic, striated-patterned content (“kiwi fruit” appearance)	Distended GB with immobile, amorphous, hyperechoic, striated-patterned content
**Nutritional management**	Homemade weight-loss diet	Commercial weight-loss diet	Same commercial weight-loss diet as Case 2	Maintenance low-fat diet

**Variables**	**Case 1**	**Case 2**	**Case 3**	**Case 4**
**Pharmacological management**	Standardized protocol	Standardized protocol	Standardized protocol + levothyroxine	Standardized protocol
**Follow up period**	30 days	60 days	30 days	90 days
**Body weight change**	-10.8%	-13.3%	-4.3%	No change
**Ultrasonographic outcome**	Complete resolution of GBM	Partial resolution of GBM	Partial resolution of GBM	Persistent GBM at 60 days; resolution of characteristic GBM ultrasonographic features with residual biliary sludge at 90 days
**Treatment status at follow-up**	Pharmacological treatment discontinued; nutritional management maintained	Pharmacological and nutritional management maintained	Pharmacological and nutritional management maintained	Pharmacological and nutritional management maintained

Legend: BW = body weight; BCS = body condition score; MMS = muscle mass score; GB = gallbladder; GBM = gallbladder mucocele; ALP = alkaline phosphatase.
